# Retraction: Resveratrol Preincubation Enhances the Therapeutic Efficacy of hUC-MSCs by Improving Cell Migration and Modulating Neuroinflammation Mediated by MAPK Signaling in a Mouse Model of Alzheimer's Disease

**DOI:** 10.3389/fncel.2021.728508

**Published:** 2021-07-23

**Authors:** Frontiers Editorial Office

The journal retracts the 27 March 2020 article cited above.

Following publication, concerns were raised regarding the integrity of the images in the published figures. The authors failed to provide a satisfactory explanation during the investigation, which was conducted in accordance with Frontiers' policies.

This retraction was approved by the Chief Editors of Frontiers in Cellular Neuroscience and the Chief Executive Editor of Frontiers. The authors did not agree to this retraction.

